# Combined treatment for a hemispheric cerebellar AVM

**DOI:** 10.3171/2020.10.FOCVID2069

**Published:** 2021-01-01

**Authors:** Bruno Loof de Amorim, Ricardo Chmelnitsky Wainberg, Juan Alberto Paz-Archila, Silvio Sarmento Lessa, Gabriela Miroslava Bustamante Vargas, Leonardo Favi Bocca, José Maria de Campos Filho, Christiane Monteiro de Siqueira Campos, Marcos Devanir Silva da Costa, Feres Chaddad-Neto

**Affiliations:** 1 Department of Neurology and Neurosurgery, Universidade Federal de São Paulo (UNIFESP/EPM); and; Divisions of 2 Neurosurgery and; 3 Neuroradiology, Hospital Beneficência Portuguesa de São Paulo, Brazil

**Keywords:** cerebellar AVM, arteriovenous malformation, endovascular treatment, microsurgical treatment

## Abstract

Posterior fossa arteriovenous malformations (AVMs) can be a challenging disease, especially those large in size. AVMs can be treated with a combination of endovascular treatment and microsurgery. Here, the authors present the case of a 16-year-old female patient with progressive dizziness and episodic syncope. The workup of the patient showed a hemispheric cerebellar AVM, Spetzler-Martin grade IV. She underwent combined treatment (endovascular and microsurgery) with no complications and cure of the malformation.

The video can be found here: https://youtu.be/rNw_Kyd76Mg

## Transcript

**0:22 Clinical Presentation.** This is a case of a 16-year-old female patient who presented progressive dizziness and episodic syncopes.

**0:28 Radiological Findings.** Further MRI investigation showed a diffuse left paramedian Spetzler-Martin grade IV cerebellar AVM,^1^ associated to a large vein of drainage.

The digital angiography demonstrated the main arterial feeders from the enlarged left PICA, which presented some aneurysms along its distal part. The right vermian branch arises from the left PICA.^2^ There were several intranidal fistulas. The venous drainage was toward the great vein of Galen and the tentorial sinuses.^3^

**1:06 Endovascular Treatment.** She was submitted to three preoperative sections of embolization with the embolic liquid PHIL, with near-total occlusion of the AVM, remaining only a partial portion on the tentorial surface. This is the final endovascular treatment cast.

PHIL is a novel liquid embolic agent composed of a nonadhesive copolymer dissolved in DMSO with an iodine component. The potential advantages of PHIL are faster plug formation, more consistent visibility, fewer artifacts in postinterventional imaging, lower volumes required of liquid agent embolic. Considering these peculiarities in our present case, the extensive nidus represents a good indication for PHIL use, as an embolic agent.

**1:59 Positioning and Opening Technique.** The patient was operated in a semisitting position, and a wide far-lateral approach was performed.^4^

**2:06 Corticectomy and Dissection of the AVM.** After dural opening, microsurgical corticectomy was started laterally to the AVM, and then surrounding the nidus. Now we see the left and right PICA with the differences between them caused by the embolic agent.

The inferior part of the AVM included the left cerebellar tonsil, and the previously tonsillomedullary and telovelotonsillar segments of PICA^5^ were cut. Because of this embolic material, PHIL, the dissection of the AVM became easier, and the soft consistency of the cast facilitate the dissection.

The dissection continues in the superior part of the AVM, at the tentorial surface of the cerebellum, once there are located the drainage veins.^6^

The anterior aspect of the AVM was then dissected^7^ and the fourth ventricle cavity exposed. Choroidal branches, feeders, and the left vermian branch be coming from the right PICA were found and cut, and the AVM was finally removed.

This is the final surgical cavity and the measure of the AVM.

**4:06 Postoperative Images and Final Considerations.** Postoperative MRI showed no residual nidus and preserved medullary parenchyma. The digital angiography confirmed the complete resection. The patient was discharged with no deficits.

## Author Contributions

Primary surgeon: Chaddad-Neto. Assistant surgeon: de Campos Filho. Editing and drafting the video and abstract: Silva da Costa, Loof de Amorim, Wainberg, Paz-Archila, Lessa, Bustamante Vargas, Campos. Critically revising the work: Silva da Costa, Bocca, Chaddad-Neto. Reviewed submitted version of the work: Silva da Costa, Bocca, Chaddad-Neto. Approved the final version of the work on behalf of all authors: Silva da Costa. Supervision: Silva da Costa, Chaddad-Neto.

## References

[1] Spetzler RF Martin NA A proposed grading system for arteriovenous malformations J Neurosurg 1986 65 4 476 483 3760956 10.3171/jns.1986.65.4.0476

[2] de Oliveira E Tedeschi E Rhoton A Peace D Microsurgical anatomy of the posterior circulation: vertebral and basilar arteries Carter L Spetzler R Neurovascular Surgery McGraw Hill 1995 25 34

[3] Yasargil M Microneurosurgery, Volume IIIA: AVM of the Brain, History, Embryology, Pathological Considerations, Hemodynamics, Diagnostic Studies, Microsurgical Anatomy Thieme Verlag 1996

[4] Chaddad-Neto F Doria-Netto HL Campos Filho JM The far-lateral craniotomy: tips and tricks Arq Neuropsiquiatr 2014 72 9 699 705 25252234 10.1590/0004-282x20140130

[5] Rhoton AL Jr. The posterior cranial fossa: microsurgical anatomy and surgical approaches Neurosurgery 2000 47 suppl 3 S5 S6 10.1097/00006123-200105000-00064 11334297

[6] Cavalheiro S Serrato-Avila JL Párraga RG Interpeduncular sulcus approach to the posterolateral pons World Neurosurg 2020 138 e795 e805 32217179 10.1016/j.wneu.2020.03.084

[7] Manduca Palmiero HO Silva da Costa MD Caramanti RL Eduardo Aparecido Chaddad-Neto F Angular and metric analysis of the neural structures in the cerebellopontine angle Br J Neurosurg 2018 32 3 250 254 29334768 10.1080/02688697.2018.1426722

